# Conceptualising factors impacting nutrition services coverage of treatment for acute malnutrition in children: an application of the *Three Delays Model* in Niger

**DOI:** 10.1017/S1368980021004286

**Published:** 2023-05

**Authors:** Stephen R Kodish, Ben GS Allen, Halidou Salou, Teresa R Schwendler, Sheila Isanaka

**Affiliations:** 1 Pennsylvania State University, Departments of Nutritional Sciences and Biobehavioral Health, 110 Chandlee Lab, University Park, PA 16802, USA; 2 Technical Support Team, GNC Technical Alliance, Action Against Hunger Canada, Toronto, Canada; 3 Epicentre Niger, Maradi, Niger; 4 Epicentre, Research Department, Paris, France; 5 Harvard T.H. Chan School of Public Health, Departments of Nutrition and Global Health and Population, Boston, MA, USA

**Keywords:** Severe acute malnutrition, Community-based management of acute malnutrition, Three delays model, Rural Niger

## Abstract

**Objective:**

The *Three Delays Model* is a conceptual model traditionally used to understand contributing factors of maternal mortality. It posits that most barriers to health services utilisation occur in relation to one of three delays: (1) Delay 1: delayed decision to seek care; (2) Delay 2: delayed arrival at health facility and (3) Delay 3: delayed provision of adequate care. We applied this model to understand why a community-based management of acute malnutrition (CMAM) services may have low coverage.

**Design:**

We conducted a Semi-Quantitative Evaluation of Access and Coverage (SQUEAC) over three phases using mixed methods to estimate programme coverage and barriers to care. In this manuscript, we present findings from fifty-one semi-structured interviews with caregivers and programme staff, as well as seventy-two structured interviews among caregivers only. Recurring themes were organised and interpreted using the *Three Delays Model*.

**Setting:**

Madaoua, Niger.

**Participants:**

Totally, 123 caregivers and CMAM program staff.

**Results:**

Overall, eleven barriers to CMAM services were identified in this setting. Five barriers contribute to Delay 1, including lack of knowledge around malnutrition and CMAM services, as well as limited family support, variable screening services and alternative treatment options. High travel costs, far distances, poor roads and competing demands were challenges associated with accessing care (Delay 2). Finally, upon arrival to health facilities, differential caregiver experiences around quality of care contributed to Delay 3.

**Conclusions:**

The *Three Delays Model* was a useful model to conceptualise the factors associated with CMAM uptake in this context, enabling implementing agencies to address specific barriers through targeted activities.

Severe acute malnutrition (SAM) among young children is a persistent and significant health challenge in low-income countries where food insecurity is prevalent and infectious diseases are endemic^(1,2)^. At any given time, approximately 6·9 % of children under 5 years of age are severely wasted, an indicator of acute malnutrition, globally^(3)^. Timely access to appropriate care for children with SAM is critical for ensuring optimal recovery and increasing survival^(3)^.

In the past decade, a new model of treatment for acute malnutrition has been shown to be cost-effective and acceptable^(4–6)^. The Community-based Management of Acute Malnutrition (CMAM) model is now implemented in over seventy countries and was designed to improve treatment coverage compared to traditional inpatient treatment. The CMAM model relies on the timely case detection at the community-level, as well as the provision of ready-to-use therapeutic foods for home use among uncomplicated cases^(7–10)^.

Despite the potential of CMAM to reach a greater proportion of SAM cases in need, worldwide coverage was just 10 % in 2018, with variability across programmes, contexts and seasons^(11)^. A 2015 review of CMAM programming in twenty-one countries found barriers to optimal coverage to consistently include a lack of caregiver awareness of malnutrition signs and symptoms, limited community awareness of CMAM services and high opportunity costs^(12)^. While an understanding of the shared barriers across CMAM programmes may begin to help programmes to improve its own coverage, application of a conceptual behavioural model to provide an understanding of how specific barriers emerge during care seeking may allow programme planners to tailor their own programmes more effectively.

As part of a Semi-Quantitative Evaluation of Access and Coverage (SQUEAC) survey conducted to evaluate CMAM program coverage in rural Niger^(13)^, we applied the *Three Delays Model* to better understand context-specific barriers to accessing treatment in this setting^(13)^. The *Three Delays Model* was first introduced as an explanatory framework to better understand the contributing factors of persistently high levels of maternal mortality in low-income settings. Given the importance of timely medical care during a pregnancy-related emergency, this model was designed with a focus on the period beginning when obstetric complications present until an outcome is realised^(14)^. Since its inception nearly 30 years ago, the model has been used widely to examine maternal mortality across country contexts^(15–18)^.

Given the importance of timely care for children with SAM, we posited that the three delays of this model: (1) Delay 1: delayed decision to seek care; (2) Delay 2: delayed arrival at health facility; and (3) Delay 3: delayed provision of adequate care, could also be a useful framework with which to understand the barriers caregivers face while accessing CMAM services.

Specifically, we aimed to first identify the context-specific factors influencing CMAM coverage in this setting of Niger and second to help identify when and what types of interventions may be targeted to improve coverage. In doing so, we also aimed to determine the utility of using the *Three Delays Model* in the context of a SQUEAC assessment.

## Methods

### Study setting

We conducted this study during the early rainy/lean season in Madaoua, Niger, a rural health district in the Tahoua region. It is located in the rural Sahel, a region characterised by seasonal food insecurity and high rates of malnutrition in children under 5 years of age^(19)^. In this district, the Ministry of Health provided SAM treatment at six outpatient and one inpatient treatment centres with support from Médecins Sans Frontières – Operational Center Barcelona. Household visits, health education and sensitisation via radio programming are also provided in eighty villages^(20)^. In 2016, CMAM program coverage in Madaoua was 26 % (95 % CI: 18 %–34 %), which is below the SPHERE standard (< 50 %)^(20)^.

### Study design and data collection methods

A SQUEAC survey was conducted with the primary objective of identifying factors that affected uptake of services and coverage. SQUEAC methodology utilises mixed data collection methods conducted over three iterative phases^(13)^.

During phase 1, we analysed routine programme data (e.g. admissions, discharges, screenings and referrals) to identify factors influencing coverage. We triangulated findings with thirty-nine semi-structured interviews with caregivers and CMAM program staff in fifteen villages across the six health centre catchment areas (see online Supplemental interview guide 1). A two-stage, criterion-based purposive sampling strategy was used to recruit interview participants. First, we purposively sampled villages to ensure a geographic representation of health catchment areas supported by Médecins Sans Frontières. Second, we recruited caregivers of SAM children as well as CMAM program staff (i.e. nurses and community health workers) to ensure multiple perspectives^(21)^.

During phase 2, we tested coverage assumptions (e.g. areas of low or high coverage) using a small area survey. This survey included searching for and enumerating cases, as well as conducting twelve semi-structured interviews with caregivers of non-covered cases in three villages (see online Supplemental interview guide 1). Finally, in phase 3, we estimated overall programme coverage utilising Bayesian techniques combining qualitative data from phases 1 and 2 and additional survey data. SAM cases in forty-six villages were found and enumerated. We then conducted seventy-two additional structured interviews with caregivers of non-covered SAM cases (see online Supplemental interview guide 2). Case finding in phases 2 and 3 was done using the active and adaptive method^(22)^.

Phase 1–3 qualitative sample sizes were determined by an estimate of the minimum number of participants needed to understand program coverage barriers and boosters based on global SQUEAC guidance that prescribes data collection within a short time frame^(13)^. All interviews were conducted by local data collectors with qualitative research experience who were fluent in Hausa and French. They participated in a 1-d refresher training on qualitative interviewing procedures, which included a team-based review of the interview guides. During fieldwork, data collectors conducted one-on-one interviews in Hausa, while writing detailed field notes that were used for textual analysis.

### Data analysis

An inductive approach was used to identify emergent themes following standardised analytic procedures for textual analysis^(23)^. Each interview form included detailed field notes, which were reviewed line by line to identify the salient boosters and barriers to CMAM program coverage based on frequency of mention. In line with standard SQUEAC procedures, analytic meetings were then held among research team members to review the emergent themes, make decisions around combining or splitting thematic categories and interpret findings in the context of this particular CMAM program. Barriers were then organised by delay using the *Three Delays Model* as the guiding analytic framework^(14)^.

## Results

We conducted thirty-nine semi-structured interviews with caregivers, health staff and community members in sixteen villages during phase 1. During phase 2, we completed twelve structured interviews with caregivers of uncovered SAM cases. Finally, during phase 3, we conducted seventy-two interviews with caregivers in forty-six villages (Table 1).

**Table 1:** Summary of qualitative methods, participant type and sample sizes by study phase

SQUEAC phase	Participant type	Qualitative method	*n*
Phase 1	Caregivers, community members, programme staff	Semi-structured interviews	39
Phase 2	Caregivers	Structured interviews	12
Phase 3	Caregivers	Structured interviews	72

Overall, eleven barriers to CMAM coverage were identified by 123 respondents. Five barriers were related to delayed decisions to seek care (Delay 1); four barriers were related to delayed arrivals to the health facility (Delay 2) and two barriers were associated with delayed provision of adequate care (Delay 3) (Table 2).

**Table 2:** Primary factors of community-based management of acute malnutrition (CMAM) coverage organised by *three delays* framework

*Three delays* description	Contributing factors of CMAM program access^*^
Delay 1: Delayed decision to seek care	• Limited caregiver knowledge on malnutrition signs/symptoms • Lack of awareness of CMAM services • Varying levels of social support to seek care (e.g. husband permission required) • Variable screening services that do not reach all villages or households • Alternative health seeking options for malnutrition (e.g. traditional health practitioner or pharmacy)
Delay 2: Delayed arrival at health facility	• High travel costs relative to household income • Poor road conditions to access health facilities • Long travel distances to health facilities • Competing demands, especially during busy agricultural seasons
Delay 3: Delayed provision of adequate care	• Insufficient health staffing leading to long wait times • Variable quality of provider–patient interactions when receiving care

* Based on salient themes derived from qualitative interviews with caregivers, community members and CMAM program staff.

### Delay 1. Delayed decision to seek care

Delayed caregiver decisions to seek care were discussed in relation to five barriers. First, when asked about the signs and symptoms of child malnutrition, some caregivers demonstrated insufficient knowledge to accurately identify SAM cases. Few participants discussed the potential impact of acute malnutrition on longer-term growth and development of children, for instance.

Second, not all community members were aware of the CMAM services available to them. And among those mothers who were aware of CMAM services, there was differential understanding towards certain programme details, for instance that children could be re-admitted for treatment more than once. In this context, CMAM details were promoted using community radio, home visits and sensitisation campaigns; however, promotions do not always reach entire communities, especially harder-to-reach villages, according to interviewees. For example, while the radio was said to be an effective channel to reach men, who are regular listeners, it is less effective for reaching women who tend to rely more heavily on interpersonal communications in this setting. To be sure, community leaders and heads of households underscored the importance of in-person visits from the CMAM program staff and the ‘community criers’ who are tasked with awareness raising. Also, we found that mothers who had had previous experiences accessing CMAM services were informal, yet important influencers to encourage uptake of services among friends and neighbors.

Third, perceived levels of social support, which varied among respondents interviewed, influenced mothers’ timeliness around decisions to seek care. While some caregivers indicated that they received encouragement from their husbands and family to seek care for their child, others indicated the contrary. Husband permission was therefore essential for most caregivers to decide to seek care and influenced by the visible condition of the child as well as the availability of financial resources for travel and described under delay 2 below.

Additionally, caregivers also described the screening and referral services by health workers to be limited in their reach and effectiveness. They explained that screenings routinely targeted some, but not all villages and were located at a central point within the community. Screenings in this health catchment area were conducted by several different non-governmental organisations using different approaches: at central village locations, door-to-door or a combination. Screenings at central locations were more common in this setting; door-to-door screenings were not carried out in most villages. Health staff confirmed that many SAM cases arrived at facility-based services without formal referrals through community screening.

Finally, community leaders and health workers both discussed the use of private pharmacies and traditional healers, as treatment alternatives to CMAM services. In this setting, traditional healers are socially acceptable and, in most cases, easier to access than health clinics in terms of both physical distance and consequently lower transport costs. In addition to cost, caregivers also explained that health-seeking decisions can be made based on the condition of the child. Oedema, specifically, was said to be more appropriately treated by traditional medicine practices than by formal medical services in this setting. Specific alternative treatments that were preferred by some caregivers included consumption of locally available plant leaves (i.e. herbal medicines), wearing a *gris-gris* (talisman to ward off evil spirts) from a healer and listening to readings from the Quran. Not all mothers preferred traditional medicine approaches though: some indicated that they preferred to treat their child with ready-to-use therapeutic foods from the health centre, especially because the services were free.

### Delay 2. Delayed arrival at health facility

Delayed arrivals at health centres offering CMAM services stemmed from four primary barriers. First, limited financial resources hindered health centre access for most caregivers, largely due to round-trip travel costs of approximately 2·50–3·30 USD, according to interviews. Mothers emphasised that even after their decision to seek care had been made, the level of support their husbands would provide still depended on available financial resources. Spousal support was most critical when initial decisions to seek care are made (Delay 1) but also influenced by the travel considerations associated with getting to a health facility. Additional travel-related challenges included long distances from villages to CMAM services and poor road conditions. During the rainy season, in particular, reliably accessing CMAM services is relative expensive and time consuming when road conditions are sub-optimal.

Finally, caregivers explained their competing demands made it difficult to find time to seek care for sick children any time throughout the year. Farming is the primary livelihood of rural Niger, and agricultural activities can require time-intensive commitments by household members, especially during planting and harvest seasons. Mothers explained the difficulties of managing both childcare and agricultural work, especially during important agricultural seasons.

### Delay 3. Delayed provision of adequate care

Upon arrival to health facilities offering CMAM services, caregivers explained two additional challenges associated with the provision of adequate care. Caregivers reported mixed experiences around the quality of medical treatment they personally experienced, as well as held differing perceptions towards care quality from other community member accounts.

Two primary themes emerged in relation to delay 3. First, some participants explained that an insufficient number of CMAM program staff at the facilities were available to provide timely services every visit. Second, caregivers described the quality of interactions with health staff to be variable. Some mothers reported generally negative interactions, including feelings of being scolded, when they presented their children with low weight to the health centre. Other respondents reported similar treatment by medical staff when treatment protocols were not followed at home. Such interactions were said to contribute to negative connotations/perceptions towards the quality of care provided. Just as many mothers reported positive experiences (e.g. free services, provision of food supplement) in our sample, however.

## Discussion

We conducted semi-structured interviews among caregivers and programme staff, as well as structured interviews with caregivers of non-covered cases as part of a larger assessment of CMAM program coverage. Findings revealed eleven barriers affecting caregiver access to CMAM services in this context. Caregivers face most barriers when they are deciding to seek care (Delay 1) and when they are travelling for medical treatment (Delay 2). Fewer barriers were associated with Delay 3: in cases when caregivers reached the desired health facility, both positive and negative experiences were reported.

Most barriers limiting CMAM access in this setting occurred within Delay 1 (delayed decision to seek care). Inadequate caregiver knowledge, variable intra-household support, low awareness of services, use of traditional medicine and limited screening/referrals were important factors that delay caregiver decisions to seek care for their children in this setting. Further investment in integrated community-based screening by health workers able to travel door-to-door across the entire health catchment area may help address inadequate knowledge, low awareness and limited screening coverage in this setting^(24)^. Other studies to understand low CMAM coverage have found a disconnect between community perceptions of malnutrition and those of the medical community^(25)^. Therefore, equipping health workers with culturally appropriate and context-specific job aides that consider literacy levels, as well as address local conceptions of malnutrition, may better empower health workers to connect with caregivers who lack household support and may be less trusting of biomedical care for child malnutrition. Coupled with community-wide awareness meetings and recurring trainings of health workers may also help to mitigate challenges around deciding to seek care for children^(26)^. Reliance only on health workers to overcome CMAM coverage challenges may be less effective than also directly involving parents as service delivery partners who may also be trained to screen their own children for malnutrition. This ‘Family-MUAC’ approach has been comparatively effective to screening by health workers in similar settings^(27–29)^.

Access to care, even after decisions to seek care are made, is still uncertain in rural Niger. Far distances and poor road conditions, coupled with competing demands and high travel costs relative to household incomes, are primary challenges to CMAM access in this setting. CMAM services typically focus on malnutrition treatment at health centres and screening/mobilisation activities at the community-level, thus necessitating travel and associated costs to reach medical treatment. For rural Nigerien households whose average household transport costs are estimated to be $40 dollars/month, a $2·50–3·00 1-time cost to reach CMAM services is not inconsequential, especially considering large family sizes with several young children who may require care per household^(30)^. The fertility rate in Niger at the time of this assessment was seven births per woman^(31)^. Access-related factors are not only important barriers to optimal care-seeking behaviour in this rural Niger context but also in many other similar low-income, rural communities throughout sub-Saharan Africa^(32)^. Various intervention strategies, such as integration of trained community health workers, and the use of monetary incentives to promote early care seeking, have been used to address these types of structural factors across contexts yet with varying levels of success^(33,34)^. For CMAM service delivery, such factors may be considered non-modifiable. However, novel intervention strategies (e.g. reimbursement of travel costs upon arrival and treatment of child with SAM; empowering community health workers to manage uncomplicated SAM cases at the household rather than facility level) to help caregivers overcome these barriers may help improve CMAM coverage in this context and others^(35)^.

Organising coverage barriers by 3 delays highlighted two additional barriers at the health centre itself: insufficient programme staff numbers causing long wait times and negative interactions with health staff. Long wait times upon arrival to a health facility are a commonly cited barrier to CMAM services across settings^(36–38)^. In this assessment, caregivers perceived shortages of staff to be the bottleneck contributing to long wait times upon arrival at the health facility. It is possible that staffing is just one of several factors contributing to long wait times. However, caregivers in this assessment did not explicitly discuss ‘quality of care’ as a barrier to health centre access but alluded to it while reflecting on long wait times and negative interactions with health workers at point of care. In other settings, negative interactions with health facility staff and low satisfaction with health services have also been found to negatively influence care seeking behaviours^(39)^. Intervention strategies to improve maternal satisfaction of care provided at health centres may be implemented across three dimensions: structure, process and outcomes^(40)^. Our findings suggest that investment in the first dimension, physical structure, by ensuring a clean, comfortable environment with adequately resourced staffing and supplies may address insufficient staff numbers and long wait times reported by respondents in this setting. Improving the process of care, which includes appropriate interpersonal interactions with medical staff, may also be appropriate given our findings. Ensuring quality standards by building in routine monitoring activities, such as direct observations of provider-patient interactions at health centres, and supportive supervision of health workers may further help to identify areas of improvement at point of care for enhancing maternal satisfaction^(41)^.

Overall, our findings reflect similar types of barriers that have been identified during SQUEAC assessments in other CMAM programs^(12,42)^. The most frequently reported barriers globally include far distances and high opportunity costs (e.g. financial constraints, competing livelihood demands)^(12,38)^ which we also found in this programme setting. The concurrence of similar barriers across settings globally may in part reflect the similar resource constraints in settings where SAM treatment is provided. Those similarities may also partially reflect the rapid nature of SQUEAC assessments which are designed to collect data over a relatively short time period using practical approaches to data collection (e.g. cross-sectional data collection, no digital recordings of qualitative interviews) that may not be well suited for capturing the level of detail needed to discern more nuanced determinants of care-seeking behaviours across socio-cultural settings. Finding a methodological balance between rapid and in-depth data collection approaches as part of SQUEAC assessments may be one area for further implementation research in the context of CMAM.

The methodological approach that we employed during this SQUEAC assessment included semi-structured qualitative interviews, which were important for revealing additional context around health behaviours in this setting. For instance, we found traditional medicine to be both an affordable and culturally acceptable alternative to CMAM services in rural Niger. Traditional medicine is typically found within cultural contexts where the underlying medical belief system may stand, at least to some extent, in contrast to modern medical approaches^(43)^. Community trust toward and uptake of biomedical approaches, like those provided in CMAM services, may be low among populations where traditional healers are among the most trusted leaders in a community. In other similar settings, locally formed Care Groups have been utilised to promote positive health and nutrition behaviours by acknowledging the importance of traditional medicine while concurrently promoting biomedical approaches such as CMAM treatment^(44)^. Care Groups may also provide a trusted channel for sensitisation and behaviour change communications where it is possible to incorporate locally understood terms for malnutrition that resonate with community members^(25)^. Using trusted communication channels, local language terms and tailored messages can be important for more effective health behaviour change^(45,46)^.

The *Three Delays Model* is a relatively simple conceptual tool that may provide a novel and useful way to organise the factors impacting CMAM coverage. Typically, SQUEAC assessments yield a lengthy list of programme coverage factors without specific consideration or analysis of the types of factors comprising the list. Organising the programme-specific barriers to understand when and where they are occurring may allow for more strategic responses to improve coverage. Using the *Three Delays Model* to conceptualise results in this assessment allowed us to understand that the majority of barriers influencing caregiver access to CMAM services were related to delay 1, when decisions to seek care are made. This understanding may be used for improving services in this setting by directing available programme resources to address key modifiable factors related to knowledge, awareness and screening within communities, specifically. Additional research that utilises the Three Delays Model for understanding CMAM coverage may aim to build in methods that allow for further understanding of the relative importance of each barrier within and between delays. Doing so within the context of a SQUEAC assessment may provide more nuanced information for practitioners who wish to improve programming but only have the resources to tackle some, not all, of the barriers to care.

This study had several limitations. First, interviews were not digitally recorded. Using digital recordings and verbatim transcriptions may have allowed for more in-depth and nuanced textual analysis. The programmatic nature of the SQUEAC methodology, which usually allows for only several days of data collection and analysis, does not lend itself to a comprehensive iterative qualitative analysis between phases. Second, interview data were not triangulated with observations, and our findings thus rely solely on participant narratives. What people say and what people do can be at odds^(47)^, particularly in health behaviour research where participants are beneficiaries of humanitarian support. Third, our interpretation of findings may have been strengthened by the incorporation of additional programme details related to dose, reach and fidelity of health promotions, specifically. Contextualising each barrier to care vis-à-vis process indicators of programme effectiveness may have allowed for more targeted recommendations to improve coverage in this setting specifically.

Despite these limitations, there are several strengths. First, the data collection team was hired locally as ‘cultural insiders’ who possessed both local language proficiency and a deep understanding of this socio-cultural context and population^(48)^. Their familiarity to the participants may have improved rapport during interviews, thus yielding richer information. Second, interview data were inductively examined by a research team member who identified emergent themes related to programme coverage, without *a priori* hypotheses that may have biased interpretations. We believe this field-level analytic approach assured credible results by following a systematic though abbreviated process. Third, findings were drawn from interviews conducted among several different types of people – a strategy called ‘participant triangulation’, which allowed for corroboration among participant narratives as well as multiple perspectives on the same situation^(49)^.

## Conclusions

Barriers identified, when organised by one of three delays, can aid practitioners in designing tailored strategies and targeted programme adjustments that address modifiable barriers at key moments during care seeking decision making. The *Three Delays Model* can serve as a useful conceptual tool to apply during standardised coverage assessments to better understand those factors influencing optimal care seeking behaviour for children with acute malnutrition and tailor programmatic response to improve health and nutrition services coverage.

## Supplementary material

For supplementary material accompanying this paper visit https://doi.org/10.1017/S1368980021004286. click here to view supplementary material
