# Supplementary material for: Conceptualising factors impacting nutrition services coverage of treatment for acute malnutrition in children: an application of the Three Delays Model in Niger
Source: Public Health Nutr. 2021 Oct 8;26(5):1074–81. doi: 10.1017/S1368980021004286 (PMC10346043; doi:10.1017/S1368980021004286)
Supplement: Supplementary file 1 [file S1368980021004286sup.zip › S1368980021004286sup001.docx]

**Formulaire d'entretien semi-directif (phase 1 – 2)**

Nom de l’enquêteur :

Nombre des mères enfants MAS:

***Formulaire/Guide pour la vérification et pondération communautaire***

**LA VERIFICATION COMMUNAUTAIRE**

**INTRODUCTION**: Nous avons parlé avec beaucoup des gens dans le district sanitaire de Madaoua au sujet de la malnutrition et la PCIMA. Nous voulons donc vérifier avec vous que nous avons bien compris et = que nos interprétations sont assez précises pour refléter la réalité. Cette discussion vous donne aussi l’occasion de nous dire si nous nous trompons sur quelque chose en particulier. Il n’a y a pas de réponse justeou fausse, nous voulons simplement vos avis sur ces points.

**1. Utilisation des services et traitement alternatif**

**Nous avons trouvé que pour traiter la malnutrition les gens recherchent les 4 traitements suivant. Est-ce que vous êtes d'accord ?? Sinon, pourquoi ?**

1. Directement au service de cas de santé
2. Préférence pour la PCIMA
3. Préférence initiale pour le guérisseur surtout quand il y a des coûts associés (ex. transport)
4. Utilisation » des pharmacies par terre » pour traiter les enfants malades

**2. Canaux d’information sur la PCIMA/malnutrition**

**Nous avons demandé aux gens comment ils trouvent l’information sur la malnutrition et le PCIMA. Nous avons trouvé 5 voies principales. Est-ce que vous êtes d'accord? Sinon, pourquoi ?**

Les gens reçoivent l’information à :

1. partir des mères qui étaient dans le programme
2. travers la radio
3. travers la sensibilisation (au CSI) par groupe ou individuelle
4. travers les visites à domicile par les relais
5. à travers le chef de village

**3. Connaissance de PCIMA et de la malnutrition par la communauté**

**Nous avons appris qu’il y a une bonne connaissance de la malnutrition mais il n’est pas rare que les mères arrivent quand l’état de l’enfant est déjà grave. Quel autre facteur peut nous aider à expliquer le retard aux soins?**

**4. Dépistage et référence (au niveau communautaire et des structures)**

**Nous avons aussi voulu déterminer quel est le système de dépistage et de référence. Nous avons trouvé 4 moyens par lesquels les enfants sont dépistés et référés. Est-ce que ceci est vrai selon votre expérience ? Sinon, pourquoi ?**

- 1. Au niveau du village l’équipe MSF fait le dépistage à un point central (ex. chez le chef de village)
  2. Au niveau du village les relais communautaires font souvent le dépistage porte-à-porte (chaque semaine)
  3. La case de santé dépiste les enfants
  4. La première fois qu’on a été dépisté est au niveau du CSI (pas dans le village)

**5. Qualité de soins**

**Nous avons aussi posé des questions pour déterminer la qualité de soins pour le PCIMA. Donc encore une fois, est-ce que vous pouvez nous dire si les points suivants sont vrais selon votre expérience ? Sinon, pourquoi ?**

1. Il y a un bon comportement, bon accueil et gentillesse par les agents de santé au niveau de CSI
2. Le personnel du centre de santé prend du temps pour faire la sensibilisation (hygiène, conseils diététiques, PECIMA) au niveau du CSI
3. La durée d’attente n’est pas longue/ou problématique
4. Le personnel du centre de santé (‘cogest’ et infirmière) estbien instruit sur la malnutrition et le PCIMA
5. S’il y a un cas d’abandon, ou non arrivé, le CRENAS fait appeler un relais pour rechercher l’accompagnant
6. Le PCIMA a une réputation très positive
7. Il y a une insuffisance de personnel au CSI pour assurer un PECIMA efficace

**6. Accès physique**

**Nos questions nous permettrons de recueillir de l’information sur l’accès physique au centre de santé. Par rapport à votre expérience, avez-vous un avis similaire ? Sinon, pourquoi ?**

1. La distance entre le village et le CSI est un problème à cause du mauvais état des routes
2. La distance entre le village et le CSI est un problème à cause du manque de moyens pour le transport
3. La distance entre le village et le CSI est un problème à cause du manque d’engin (ex. moto)
4. Mon mari me donne l’agent pour payer les véhicule afin d’aller au centre.

**7. Les aspects intra-communautaires**

**Nous avons étudié aussi comment les relations au sein de la communauté peuvent aider ou bien, empêcher, l’accès aux soins. Avez-vous la même compréhension et la même expérience ? Sinon, pour quoi ?**

1. La maman à l’appui de son mari, la famille ou la communauté et ils encouragent pour aller au centre de traitement, y compris avec la dotation de l’argent pour payer le transport
2. La maman peut s’organiser avec la famille (pour les travaux domestiques) pour assurer qu’elle est disponible pour aller au CRENAS
3. Il existe parfois le refus du mari et aussi de la famille
4. Souvent les femmes se moquent des mamans avec des enfants malnutris
5. Les mères abandonnent à cause de la migration (surtout les Touareg et les Peulhs)
6. Il y a des absences à cause des mariages/baptêmes/cérémonies
7. Manque de temps de la part des mamans
8. Si le chef de village recommande quelque chose (ex. le PCIMA) on le fait
