# Supplementary material for: Conceptualising factors impacting nutrition services coverage of treatment for acute malnutrition in children: an application of the Three Delays Model in Niger
Source: Public Health Nutr. 2021 Oct 8;26(5):1074–81. doi: 10.1017/S1368980021004286 (PMC10346043; doi:10.1017/S1368980021004286)
Supplement: Supplementary file 1 [file S1368980021004286sup.zip › S1368980021004286sup002.docx]

**Formulaire d'entretien structuré (phase 3)**

| **Est-ce que vous habitez dans ce village/quartier ? :** ☐ Oui  ☐ Non | | | **Village___________________** | |
| --- | --- | --- | --- | --- |
| **Nom de l’enquêteur: ______________________________________** | | | **Date : le _________ juillet 2016** | |
| **Nom de la mère : __________________________________________________** | | **CSI référé : __________________________** | | |
| **Nom de l’enfant : _________________________________________________________** | | | **Prénom de l’enfant : ___________________________** | |
| **Sexe : M F** | **Age:________mois** | |  | |
| **Œdème (0, +, ++, +++) : ________ Poids (kg) : ___ ___ , ___ ___kg. Taille: ___ ___ ___ cm. Couché 🞎 Debout 🞎 PB ___ ___ ___ mm** | | | | |
| **1. Est-ce que vous pensez que votre enfant est malade ?** ☐ Oui  ☐ Non | | | | |
| Si oui, de quelle maladie souffre votre enfant ? **______________________________________________** | | | |  |

| **2. Est-ce que vous pensez que votre enfant est malnutri ?** ☐ Oui ☐ Non | |
| --- | --- |
| Si oui, décrivez ce qu’elle a dit avec les mots exacts:  **____________________________________________________________________________________________________________________________________________________________________________________________________________________________________________________________________________________________________________________________________________________________________________________________________________________________________________________________** | |
| **3. Est-ce que vous savez où on peut soigner les enfants malnutris ?** ☐ Oui ☐ Non | |
| Quel est le type de traitement?:  🞎 Guérisseur  🞎 Pharmacie  🞎 Case de santé  🞎 CRENAS 🡪 Quel est le centre de santé plus proche qui offrant ce programme ?**_____________________________________________________** | |
| **4. Pourquoi n’avez-vous pas amené votre enfant au CSI pour bénéficier du traitement ?** *(la réponse de la mère PAS l’avis de l’enquêteur)* | |
| **_____________________________________________________________________________________________________________________________________________________________________________________________________________________________________________________________________________________________________________________________________________________________________________________________________________________________________________________________________________________________________________________________________________________________________________________________________________________________________________________________________________________________________________________** | |
| **5. A-t-il déjà été inscrit dans le programme de la prise en charge de la malnutrition aigüe sévère (MAS) au CRENAS ?**  ☐ Oui ☐ Non | |
| ☐ Si oui; quel était le résultat de ce traitement? | |
| ☐ 1. Abandon ; quand ? **__________________________** et pourquoi ? **_______________________________________** | |
| ☐ 2. Enfant sorti guéri ; quand ? **_____** | ☐ 3. Enfant sorti  non guéri ; quand ? **_____** |
| ☐ 4. Autre raison : **____________________________________________________________________________________** | |
|  | |
| **6. Est-ce que l’enfant est en cours de traitement dans un autre programme pour la malnutrition ?**  ☐ Oui CRENAM avec le CSB (bouillie) Oui CRENAM avec le *PlumpySup* (sachet jaune) ☐ Non  **[si oui demandez à voir le CSB ou les sachets de PlumpySup]** | |
|  | |
| **Commentaires additionnels :** | |

**Remerciez la maman, référez l’enfant et demandez si elle connait d’autres enfants malad**
